# Characterisation of human kallikrein 6/protease M expression in ovarian cancer

**DOI:** 10.1038/sj.bjc.6602041

**Published:** 2004-08-10

**Authors:** X Ni, W Zhang, K-C Huang, Y Wang, S-K Ng, S C Mok, R S Berkowitz, S-W Ng

**Affiliations:** 1 Laboratory of Gynecologic Oncology, Division of Gynecologic Oncology, Brigham and Women's Hospital, Boston, MA 02115, USA; 2 Gillette Center For Women's Cancer, Dana-Farber Harvard Cancer Center, Boston, MA 02115, USA; 3 Harvard Medical School, Boston, MA 02115, USA; 4 Department of Biological Chemistry and Molecular Pharmacology, Harvard Medical School, Boston, MA 02115, USA; 5 Department of Mathematics, Centre of Statistics, University of Queensland, St Lucia, Brisbane, Australia

**Keywords:** serine protease, monoclonal antibody, ovarian cancer, biomarker, gene amplification

## Abstract

Kallikrein 6 (hK6, also known as protease M/zyme/neurosin) is a member of the human kallikrein gene family. We have previously cloned the cDNA for this gene by differential display and shown the overexpression of the mRNA in breast and ovarian primary tumour tissues and cell lines. To thoroughly characterise the expression of this kallikrein in ovarian cancer, we have developed a novel monoclonal antibody specific to hK6 and employed it in immunohistochemistry with a wide range of ovarian tumour samples. The expression was found elevated in 67 of 80 cases of ovarian tumour samples and there was a significant difference in the expression levels between normal and benign ovarian tissues and the borderline and invasive tumours (*P*<0.001). There was no difference of expression level between different subtypes of tumours. More significantly, high level of kallikrein 6 expression was found in many early-stage and low-grade tumours, and elevated hK6 proteins were found in benign epithelia coexisting with borderline and invasive tissues, suggesting that overexpression of hK6 is an early phenomenon in the development of ovarian cancer. Quantitative real-time reverse transcription–polymerase chain reactions also showed elevated kallikrein 6 mRNA expression in ovarian tumours. Genomic Southern analysis of 19 ovarian tumour samples suggested that gene amplification is one mechanism for the overexpression of hK6 in ovarian cancer.

Ovarian cancer is the fourth most common form of cancer in females in the United States and accounts for more than half of the deaths due to gynaecological malignancy (Ozols, 2002). Ovarian cancer of epithelial origin, which constitutes more than 90% of the disease, is the most lethal among all gynaecological malignancies, with a 5-year survival rate of only 20% (Bast *et al*, 1990, pp 265–275; Taylor and Schwartz, 1994; Schwartz and Taylor, 1995). The poor prognosis is mainly due to the lack of symptoms at the early stage of disease. At the time of diagnosis about 70% of patients have cancer cells already spread to the pelvic and abdominal viscera or developed distant metastasis (Stage III/IV) and are rarely curable. In contrast, the survival rate will exceed 90% at 5 years if the disease is detected in Stage I, when tumours are confined to the ovaries (Junor *et al*, 1994). Therefore, early detection and early intervention is critical to improve the clinical outcome of ovarian cancer patients.

Ovarian carcinogenesis is believed to be a multistep process. To investigate the features of the earliest form of ovarian carcinoma, extensive pathological studies of grossly normal ovarian samples have revealed that high-grade serous ovarian carcinomas might develop *de novo* from surface epithelium and its inclusions, as well as from endosalpingeal tissues in the cortical stroma (Ryuko *et al*, 1992; Bell and Scully, 1994). The coexistence of cytologically benign, borderline and malignant epithelium in some ovarian carcinomas and evidence of areas of histologic transition from benign to borderline or to malignant epithelium support the contention that many low-grade ovarian carcinomas, particularly those of the mucinous type, may arise from pre-existing benign or borderline lesions (Powell *et al*, 1992; Scully *et al*, 1992, pp 139–144). Despite these pathological findings, the molecular basis for the pathogenesis of epithelial ovarian tumours is largely unknown. Discovery of biomarkers that are present early in the abnormal endosalpingeal tissues or in benign or borderline lesions coexisting with malignant carcinomas may further our understanding of the mechanism of early ovarian carcinogenesis.

We have previously identified by differential display a cDNA sequence that is highly expressed in primary breast and ovarian tumour tissues and cell lines (Anisowicz *et al*, 1996). The encoded protein, which originally was named as protease M, shows strong homology to the human kallikrein (hK) family proteins (for a review see Schachter, 1980; Yousef and Diamandis, 2001) and has a revised nomenclature of kallikrein 6 (*KLK6* for the gene and hK6 for the encoded protein). There are at least 15 human kallikrein genes that are co-localised as a cluster within a 300-kb region at chromosome 19q13.3–13.4. They also share significant similar genomic organisation and homology at both the nucleotide and amino-acid levels (Yousef and Diamandis, 2001). All genes encode for putative serine proteases with conserved signal peptide sequence for secretion and catalytic triad residues in the appropriate positions. Of them, kallikrein 3 (hK3), or more commonly named as prostate-specific antigen (PSA), has gained prominence as the most valuable tumour marker and is currently used widely for the diagnosis, monitoring, and population screening for prostate cancer (Catalona *et al*, 1991; Mettlin *et al*, 1993). hK3 has also been reported to cleave insulin-like growth factor-binding protein-3, resulting in increased availability of insulin-like growth factor. This suggests that hK3 enzymatic activity may promote proliferation, migration, and metastasis of prostate cancer cells (Cohen *et al*, 1992). It will be of interest to determine the functions and the potential of serum biomarker development for the other members of the kallikrein family. Indeed, recent studies have shown that the expression of many kallikreins including hK4, hK5, hK6, hK7, and hK8 has emerged as being related to breast, ovarian, and other human cancers (Underwood *et al*, 1999; Diamandis *et al*, 2000; Kim *et al*, 2001; Yousef *et al*, 2003). Further studies of these kallikreins in ovarian cancer and the development of detection tools may facilitate better understanding of this family of proteases in ovarian cancer and improve the prognosis of ovarian cancer patients.

In order to characterise the expression pattern of hK6 in different subtypes and stages of ovarian tumour tissues and to develop a tool for the potential use of measuring hK6 levels in patient serum, we have established a novel monoclonal antibody that has high specificity and reactivity to hK6. Here we report the utility of this monoclonal antibody in determining the expression pattern of different subtypes and stages of ovarian tumour tissues. In conjunction with the data of mRNA expression, we found that hK6 is highly expressed in various subtypes of ovarian tumour tissues, and is also present in early-stage tumours. We also observed high expression of hK6 in the apparently benign epithelia coexisting with borderline and invasive tumours in some samples. The elevated expression of hK6 in early-stage and low-grade ovarian tumours may suggest that upregulation of this protein may be an early event during ovarian cancer development and hK6 may have potential use as biomarkers for the early detection of ovarian cancer. The presence of high-quality monoclonal antibodies will be beneficial for the development of a standard screening method for large-scale population screening. We have also performed genomic Southern analysis to determine if gene amplification is one mechanism for the overexpression of hK6 in ovarian tumours.

## MATERIALS AND METHODS

### Biologic specimens

All patient-derived biologic specimens were collected and archived under protocols approved by the Human Subjects Committee of the Brigham and Women's Hospital, Boston, MA, USA. Ovarian tissues and cells were freshly collected from women undergoing surgery at the Brigham and Women's Hospital for a diagnosis of primary ovarian cancer or from control subjects having hysterectomy and oophorectomy for benign disease. All tumour tissues were collected from the primary ovarian sites and were confirmed histologically. Cultures of normal human ovarian surface epithelial (HOSE) cells were established by scraping the surface of the ovary and growing the recovered cells in a mixture of medium 199 and MCDB105 medium supplemented with 10% fetal calf serum (Sigma Chemical Co., St. Louis, MO, USA) as described previously (Tsao *et al*, 1995). For fresh-frozen sections, fresh specimens were embedded in Tissue Tek OCT medium (Miles, Inc., Indianna, USA), snap-frozen in liquid nitrogen, and stored at −80°C until use.

### Isolation of monoclonal antibodies specific to hK6

cDNA for hK6 was amplified using RT–PCR reaction with the primers 5′-CTGGAATTCTTGGTGCATGGCGGACCC-3′ and 5′-CTGTCTAGATCACTTGGCCTGAATGGTTTT-3′. The fragment was restricted with *Eco*RI and *Xba*I and subcloned into the pMAL-c2X vector DNA (New England BioLabs, Beverly, MA, USA) that has been restricted with the same enzymes. The resulting construct was transformed into the *Escherichia coli* strain TB1. The procedures for the induction of maltose-binding protein (MBP)-hK6 recombinant fusion protein synthesis by isopropyl thiogalactoside (IPTG), preparation of crude extracts, and the purification of the recombinant protein over the amylose resin column were according to the manufacturer's recommendation. Kallikrein 6 protein was released from the fusion protein by Factor Xa digestion and purified by SDS–polyacrylamide gel electrophoresis. The antigen was injected into five female Balb/c mice in a series of three injections given at 3-week intervals. The first consisted of 75 *μ*g per mouse in Freunds complete adjuvant, while the second and third injections were 50 *μ*g per mouse in incomplete Freunds adjuvant. Mice were boosted with 50 *μ*g of protein 4 days prior to fusion.

The spleen cells were fused with NS-1/Ag3 myeloma cells using polyethylene glycol, and plated out in HAT-selective growth medium. After 10 days, supernatants from each well were assayed by enzyme-linked immunosorbent assay (ELISA) and colonies showing reactivity were also tested by Western blotting. Specific antibody-secreting hybridomas were picked and transferred to separate wells in a 24-well plate and the specificity was further confirmed by ELISA and Western blotting. Positive lines were subsequently cloned by double-dilution and reassayed. Positive clones were expanded and the IgG subclasses were determined by ImmunoPure isotyping kit from Pierce.

### *In situ* immunohistochemistry

For *in situ* immunohistochemistry, 7 *μ*M sections were cut from the paraffin archived tissues and mounted on Superfrost/Plus microscopic slides (Fisher Scientific, Pittsburgh, PA, USA), and incubated at 50°C overnight. They were deparaffinised in xylene and rehydrated in graded ethanol. For antigen unmasking, sections were immersed in antigen unmasking solution (Vector Lab, Inc., Burlingame, CA, USA) and boiled in microwave oven for 10 min. The tissues sections were then washed in phosphate-buffered saline (PBS) and quenched in 0.2% H_2_O_2_ for 20 min. The sections were washed in PBS for 20 min, incubated with normal horse blocking serum for 20 min, and subsequently incubated overnight with anti-hK6 antibody. After incubation, the sections were washed in PBS for 10 min, incubated with diluted biotinylated secondary horse anti-mouse antibody for 30 min, and washed again in PBS for 10 min. After washing, the sections were incubated with VECTASTAIN Elite ABC reagent (Vector Inc.) for 30 min, washed in PBS for 10 min, incubated in diaminobenzidine (DAB) solution for 5 min, and washed in water for 10 min. The sections were counterstained with haematoxylin, dehydrated with an ascending series of alcohol, cleared in xylene, and mounted in Permount (Fisher Scientific). The specificity of the staining was confirmed by preabsorbing the antibody with the purified hK6 protein for 2 h at 37°C before applying to the sections. The results of immunohistochemistry were quantified using a semiquantitative scoring system (Yiu *et al*, 2001). The weighted score is obtained by multiplying the staining intensity score (3+, strong positive stain in most cells; 2+, moderate stain in cells; 1+, weak stain in cells; 0, no evidence of stain) and score for the percentage of positive cells (3+, most of cells stained; 2+, half of cells stained; 1+, few cells stained; 0, no cells stained). Representative photmicrographs were recorded by a digital camera (Optronic, Inc., Muskogee, OK, USA).

### Laser capture microdissection

Tissues stored in Tissue Tek OCT medium at −80°C were sectioned at 7 *μ*M in a cryostat (Leica, Inc., Northvale, NJ, USA). Sections were mounted on uncoated glass slides and immediately fixed in 70 and 50% ethanol for 30 s each, stained with haematoxylin–eosin, dehydrated in alcohol solution of increasing concentration, and cleared in xylene for 5 min. After being air-dried for 3 min, the sections were laser microdissected using the PixCell II apparatus (Arcturus, Inc., Mountain View, CA, USA).

### Quantitative real-time reverse transcription–polymerase chain reaction

Quantitative real-time reverse transcription–polymerase chain reaction (qRT–PCR) was used to quantify the mRNA levels of kallikrein 6 in the tissue samples. Frozen tumour tissues were pulverised on dry ice and total RNA was isolated using TRIzol reagent (Invitrogen, Carlsbad, CA, USA). For LCM dissected materials, RNA was extracted using a StrataPrep Total RNA miniprep kit (Stratagene, La Jolla, CA, USA). cDNA synthesis was performed with 50 ng of RNA samples using the TaqMan reverse transcription reagent kit (Perkin-Elmer, Wellesley, MA, USA), and quantitative PCR reactions were performed using an SYBR Green I kit, which contained 2 *μ*l of cDNA, 1 × SYBR PCR buffer, 3 mM MgCl_2_, 0.8 mM dNTP, and 0.025 U *μ*l^−1^ AmpliTaq Gold (PE Applied Biosystems, Inc., Framingham, MA, USA). Real-time PCR primers for kallikrein 6 (forward primer: 5′-TCCTTCCCCCGACTCAAGAAT-3′; reverse primer: 5′-TCCGCCATGCACCAACTTA-3′) were designed using the PrimerExpress software (Perkin Elmer). Another PCR reaction using a set of primers for the housekeeping gene, cyclophillin A, was used to normalise for variances in input cDNA. The reactions were performed in an ABI PRISM 5700 Sequence Detector (PE Applied Biosystems, Inc.) with denaturation for 10 min at 95°C followed by 40 PCR cycles of denaturation at 95°C for 15 s and annealing/extension at 60°C for 1 min. The threshold cycle (*C*_T_) value for each reaction, reflecting the number of PCR cycles needed to give exponential amplification of kallikrein 6 amplicon, and the relative level of kallikrein 6 for each sample was calculated as described (Huang *et al*, 2002). At least duplicated PCR reactions were performed and the values averaged for each sample.

### Statistical analysis

All analyses were performed using MINITAB version 13 (MINITAB Inc.). One-way ANOVA is adopted to test whether the mean kallikrein 6 expression levels vary depending on the subtype, diagnosis, grade, and stage respectively. The relative measures of kallikrein 6 mRNA level were compared using log-tranformed values. When an overall F-test was significant in the ANOVA, Fisher's pairwise comparisons were used to compare the mean kallikrein 6 levels among groups.

### Establishment of SKOV3 cells that overexpress hK6 and immunostaining

Full-length cDNA encoding kallikrein 6 was produced by reverse transcription polymerase chain reaction using the primer set (Forward primer: 5′-GGCGGACAAAGCCCGATTGTTCC-3′, reverse primer: 5′-GATCTCGAGTCAATCGTGATGGTGATGGTGATGCTTGGCC TGAATGGTTTTTTGGATCC-3′). The PCR product was first cloned into the TOPO TA cloning vector, pCRII-TOPO (Invitrogen). The cDNA was then restricted from the vector by *Eco*RI and *Not*I digestion and cloned into Invitrogen T-REx mammalian inducible vector pcDNA6/TO/mycHis A at *Eco*RI and *No*tI sites. The resulting construct was transfected into an SKOV3 cell line that also expresses regulator protein TetR from an integrated plasmid, pcDNA6/TR (Invitrogen). Positive sublines were selected using antibiotics blasticidin and zeocin as described in the manufacturer′s protocol. For immunstaining, the SKOV3 cell line that harboring the hK6-expressing construct was induced in the presence of 1 *μ*M of tetracycline, while the control is the same cell line treated with the solvent vehicle. These together with OVCA 429 and the immortalised HOSE cell line were fixed by adding 4% paraformaldehyde. The fixed cells were washed twice in PBS for 20 min, permeabilised in 0.2% Triton X-100 in PBS, washed again, incubated with normal horse blocking serum for 20 min, and subsequently incubated with anti-hK6 antibody at room temperature for 1 h. After incubation, the cells were washed in PBS for 10 min, incubated with diluted biotinylated secondary horse anti-mouse antibody for 30 min, and washed again in PBS for 10 min. After washing, colour development was performed using the VECTASTAIN Elite ABC Kit (Vector Inc.) as described above.

### Genomic Southern analysis

Frozen tumour tissues were pulverised on dry ice and genomic DNA was isolated according to the standard protocol (Ausubel *et al*, 1988). For Southern blot analysis, 5 *μ*g of genomic DNA was restriction digested with *Bam*HI and electrophoretically resolved on a 1% agarose gel. The genomic DNA was denatured and neutralised, transferred onto nylon membrane (Amersham, Piscataway, NJ, USA) according to the manufacturer's recommendation. Full-length kallikrein 6 cDNA was labelled with *α*-[^32^P]dCTP using the multiprime labelling kit (Roche Molecular Diagnostics, Indianapolis, IN, USA) and hybridised to the membrane using the standard protocol (Ausubel *et al*, 1988). Genomic DNA extracted from normal HOSE primary cultures was used as control. Stringent hybridisation and wash conditions were employed to ensure no cross-hybridisation of the probe to other kallikrein genes. Hybridisation signals were detected by autoradiography and quantified by a Bio-Rad BGS-700 densitometer.

### Quantitative real-time polymerase chain reaction for genomic DNA

Quantitative real-time polymerase chain reaction (qPCR) was used to quantify the gene copy number of kallikrein 6 gene for the tissue samples. In total, 10 ng of tissue genomic DNA and two kallikrein 6 primer sets (5′-end primer set: 5′-ACCCTCCAGCCCATACCAAC3′ and 5′-ACATGGGAAACCACAGGCA-3′; and 3′-end primer set: 5′-GGACGCAAAGAAAGGGCAG-3′ and 5′-CCACCTCGTGTCTTGAGGACA-3′) were used in the genomic qPCR reactions. Quantitative PCR reactions were performed using an SYBR Green I kit as mentioned for qRT–PCR. Primer sets for two single-copy genes, phosphatidylserine decarboxylase (5′-AGCAGAGCCACACAGCCTTC-3′ and 5′-GGTGAATGTGGGAACGGAAA-3′) and DNA topoisomerase II beta (5′-CCTCATCCTTAGAGGCCCCA-3′ and 5′-GAGACCTAACCGGGAATCCG-3′) were used to normalise the input DNA for different samples.

## RESULTS

### Isolation and characterisation of monoclonal antibodies specific to kallikrein 6

**Figure 1:**
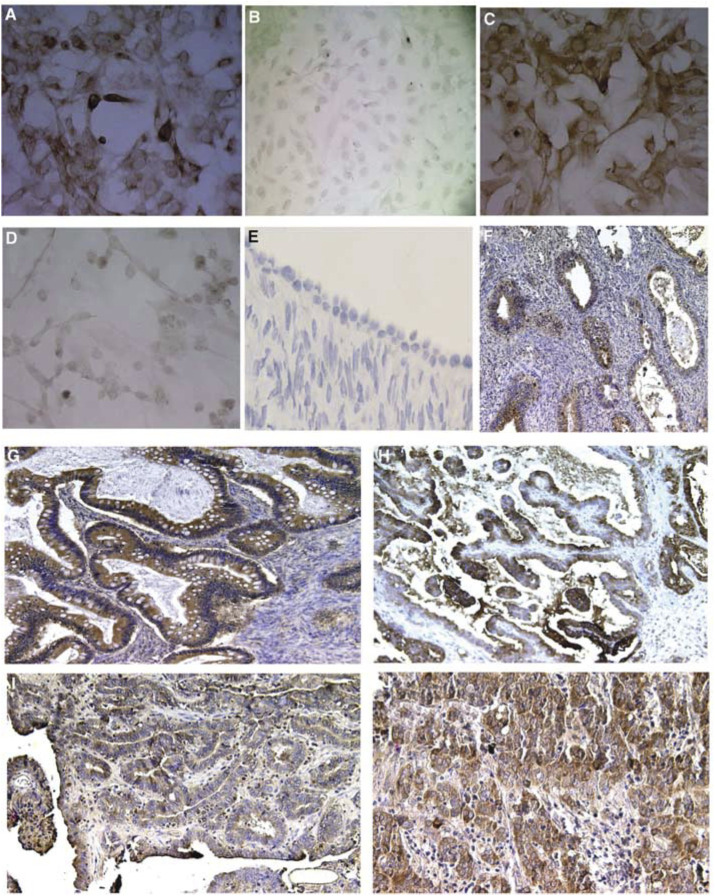
Use of monoclonal antibody 2D4 in immunostaining and immunohistochemistry. Immunostaining of (**A**) tetracycline-induced hK6 in SKOV3 cell line; (**B**) the same cell line without tetracycline induction; (**C**) OVCA429 ovarian carcinoma cell line; (**D**) immortalised normal HOSE cells. Immunohistochemical staining for hK6 in (**E**) normal ovary; (**F**) endometrioid; (**G**) mucinous; (**H**) stage I clear cell; (**I**) stage I serous; and (**J**) stage III serous ovarian tumour tissues. Immunopositive cells were stained brown in the cytoplasm. To highlight the tumour cells, the slides were counterstained with haematoxylin (purple).

In order to generate specific antibody for hK6, full-length cDNA for *KLK6* gene was cloned into (MBP) encoding pMAL-c2X vector as described in Materials and Methods. Fusion proteins were purified from the lysates of transformed *E. coli*. The hK6 protein was released from the fusion by Factor Xa digestion and used in the immunisation of mice. Spleen cells harvested from one of the immunised mouse were fused with the myeloma cell line NS-1/Ag3. After screening for more than 1000 clones, one of the clones, 2D4, showed very high reactivity and specificity to hK6 protein. 2D4 does not crossreact with trypsin and other kallikrein members such as hK1 and hK3 (data not shown). As shown in Figure 1

, the antibody stained positively the cytoplasm of an SKOV3 cell line that was induced to express hK6 by tetracycline (Figure 1A) but not in the uninduced cells (Figure 1B). The antibody also recognised the endogenous hK6 proteins present in the OVCA429 cell line (Figure 1C). The immortalised normal HOSE cells only stained negatively in the assay (Figure 1D). The IgG subclass of 2D4 was determined to be IgG2b by ImmunoPure isotyping kit from Pierce.

### Characterisation of hK6 expression in normal and tumour ovarian tissues

We employed 2D4 in immunohistochemical studies to determine the expression levels of hK6 in paraffin block sections of three normal ovarian tissues, nine benign tissues, 18 borderline and 62 invasive ovarian tumours. The mean immunostaining scores in tissue sections from healthy ovary, benign ovarian tumour, borderline ovarian tumour, and invasive ovarian cancer were 0.0, 1.83, 3.42, and 3.71, respectively (Table 1

**Table 1:** Expression of hK6 in relation to histopathologic characteristics by immunohistochemical analysis

**Characteristics**	**No. of patients**	**Mean of scores**	***P*-value**
All patients	92	3.35	
*Diagnostic category*			
Healthy	3	0.00	<0.001
Benign	9	1.83	
Borderline	18	3.42	
Invasive	62	3.71	
			
*Histology of cancer*			
Serous	44	3.44	0.205
Mucinous	16	3.94	
Endometrioid	9	3.11	
Clear Cell	4	5.00	
Mixed	7	4.14	
			
*Tumour differentiation*			
Borderline	18	3.42	0.792
Grade 1	23	3.46	
Grade 2	7	4.07	
Grade 3	29	3.64	
			
*Stage*			
I	24	3.48	0.965
II	11	3.36	
III	31	3.61	
IV	5	3.70	

). The overall F-test for the diagnostic groups was statistically significant (*P*<0.001), indicating that mean immunostaining scores varies between diagnostic groups. The Fisher's pairwise comparison procedure was employed to compare the mean immunostaining scores at 5% level. The result shows that the healthy and benign tumours have significantly lower scores than the borderline and invasive tumours. Within the cancer (borderline and invasive) groups, there was no significant difference among histologic groups, as well as among different grades and stages (Table 1). Figures 1(E through J) show representative results of the hK6 staining in normal ovarian epithelium, stage I and stage III serous tumours, as well as mucinous, endometrioid, and clear cell subtypes of ovarian tumour tissues. While there was no positive staining for the normal ovarian epithelial and stroma cells, hK6 immunoreactivity was observed in the cellular membrane and cytoplasm of tumour cells in the cancer groups.

**Figure 2:**
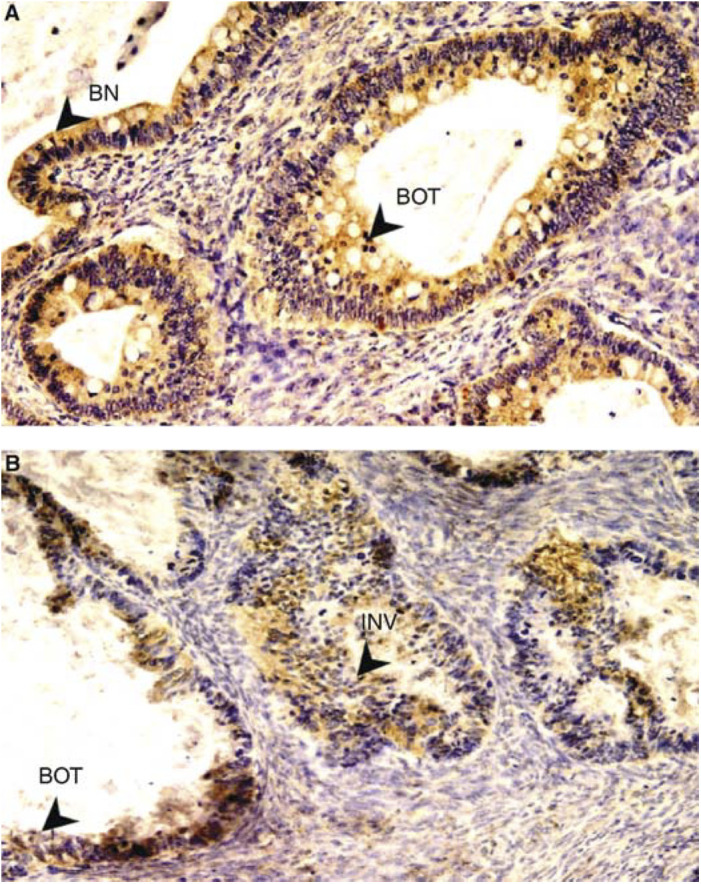
Elevated expression of hK6 in benign and borderline lesions of mucinous ovarian tumours. Positive 2D4 staining in immunohistochemistry of (**A**) a mucinous case, showing both single stratified layer of benign (BN) epithelium and borderline (BOT) tumour component with pleomorphism; (**B**) a mucinous tumour showing positive 2D4 staining in both borderline (BOT) cells and invasive (INV) tumour components.

In particular, we observed that in some mucinous tumour samples, there was strong hK6 staining in some apparently benign epithelia similar to the coexisting borderline tumours (Figure 2A

), or in borderline tumours coexisting with invasive tumours (Figure 2B). As it is believed that mucinous ovarian carcinomas may arise from pre-existing benign or borderline lesions, it is very likely that elevated hK6 expression coincides with the early stages of ovarian cancer development.

Besides the evaluation of protein expression, we also determined kallikrein 6 mRNA levels between healthy human HOSE primary cell cultures and borderline and invasive tumours. For the borderline and invasive tumour samples other than serous subtype, tumour cells were microdissected from frozen sections by Laser Capture Microdissection (LCM) and total RNA was extracted from the captured tumour cells. The results in Table 2

**Table 2:** Human kallikrein 6 mRNA expression in ovarian tissues

**Characteristics**	**No. of patients**	**Mean of expression**	***P* value**
All patients	59	3.61	
*Diagnostic category*			
Healthy	7	−0.76	
Borderline	5	4.48	<0.001
Invasive	46	4.05	
			
*Histology of cancer*			
Serous	35	4.61	
Mucinous	6	3.00	
Endometrioid	5	4.67	0.054
Clear cell	2	4.42	
Mixed	4	1.64	
			
*Tumour differentiation*			
Borderline	4	4.78	
Grade 1	5	4.04	
Grade 2	9	3.62	0.819
Grade 3	33	4.26	
			
*Stage*			
I	10	4.21	
II	4	3.16	
III	28	4.26	0.757
IV	7	3.66	

Expression is determined by quantitative real-time reverse transcription–PCR. For statistical analysis, natural logarithm was taken for the expression levels of KLK6 in tumour cells relative to normal HOSE cells.

confirmed the significant differences in kallikrein 6 mRNA expression between normal HOSE primary cell cultures and tumour tissues (*P*<0.001). For comparisons between different subtypes of tumours, the F-test in the ANOVA was marginally significant (*P*=0.054), indicating that the mean expression levels were not the same for different subtypes of tumours. The result of the Fisher's pairwise comparison further indicated that mixed tumours had significantly lower mRNA expression levels than other types of tumours. It can be seen from Table 2 that the mean kallikrein 6 mRNA levels are not significantly different among various stages and grades of borderline and invasive tumour samples.

### Characterisation of kallikrein 6 gene copy number in ovarian carcinomas

**Figure 3:**
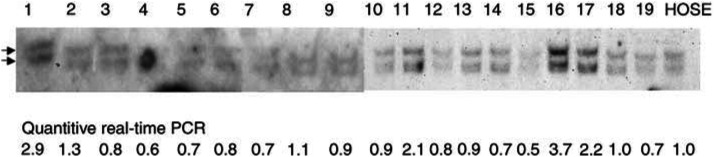
Genomic Southern analysis and quantitative real-time PCR of tumour DNA. Equal amounts of 19 tumour DNA were restricted with *Bam*HI and electrophoresed on an agarose gel and Southern blot analysis was performed using the *KLK6* cDNA probe. The 5- and 3.2-kb bands of the *KLK6* gene are marked by arrows. Genomic DNA of a normal ovarian epithelial primary culture (HOSE) was used as control. Results of the quantitative real-time PCR of the tumour samples relative to the normal HOSE cells are shown at the bottom.

In order to determine if gene amplification is one mechanism in causing elevated hK6 expression, we have performed genomic Southern analysis for 19 ovarian carcinoma samples. The results are presented in Figure 3

. Four out of 19 ovarian carcinomas exhibited higher copy number of kallikrein 6 gene. We also performed quantitative real-time PCR with the genomic DNA samples and the results were consistent with those of the genomic Southern analysis (Figure 3).

## DISCUSSION

As most ovarian cancer patients are diagnosed only at a late stage and have a dismal overall survival rate, there is a paramount need for better early detection methods. Although a number of tumour markers have been identified (Bast *et al*, 1987; Berek and Bast, 1995; Mackey and Creasman, 1995; Chen and Karlan, 1998), a useful screening marker for ovarian cancer has not yet been clearly established. The most widely used marker, CA125, has shown merit in pilot screening studies and ovarian cancer management, but the sensitivity for early-stage disease before clinical detection remains questionable (Bast *et al*, 1998).

We have previously identified by differential display a cDNA that is highly expressed in ovarian tumour (Anisowicz *et al*, 1996). Sequence analysis has shown that this novel protein belongs to the human kallikrein protein family. One member of the kallikrein family, prostate-specific antigen (PSA), has been successfully used in the diagnosis and clinical management of prostate cancer (Catalona *et al*, 1991; Mettlin *et al*, 1993). The high expression of hK6 in ovarian tumour cells and its secretory nature suggests that this kallikrein may have potential as a novel biomarker for ovarian cancer detection. To develop a useful screening tool and to examine the clinical relevance of hK6, we have developed a novel monoclonal antibody that has high specificity and reactivity to hK6. This antibody does not react with other kallikrein members and it may be very valuable in evaluating the clinical potential of hK6.

The employment of the monoclonal antibody in immunohistochemistry of archived ovarian paraffin sections has shown the significant overexpression of hK6 proteins in ovarian tumour cells compared with normal ovarian epithelium and benign diseases. There was no significant difference in hK6 expression among different subtypes, grades, and stages of ovarian cancer groups. However, we saw a slightly higher proportion of clear cell samples that overexpress hK6 (Table 1). High expression of hK6 was also observed in the borderline and early-stage invasive samples. In particular, we observed that in some mucinous tumour samples, there was strong hK6 staining in some apparently benign epithelia similar to the coexisting borderline tumours (Figure 2A), or in borderline tumours coexisting with invasive tumours (Figure 2B). As it is believed that mucinous ovarian carcinomas may arise from pre-existing benign or borderline lesions (Powell *et al*, 1992; Scully *et al*, 1992, pp 139–144), upregulation of hK6 expression may be an early event of cancer development and hK6 may have potential as a novel biomarker for early detection of ovarian cancer. In addition, mRNA levels were mostly consistent with the protein levels, suggesting that transcriptional regulation may be a mechanism that regulates the expression of hK6. Interestingly, many of the kallikrein genes are coregulated by steroid hormones (Young *et al*, 1995), and recent analysis of the genomic sequence of the kallikrein gene cluster has identified a minisatellite element that is present only in this region of chromosome 19 (Yousef *et al*, 2001). Studies of the significance of this element and other potential sequences that regulate the expression of kallikrein genes may reveal the early events of kallikrein upregulation, particularly in relation to ovarian cancer development. Nevertheless, our genomic Southern analysis and genomic quantitative real-time PCR data have indicated amplification of kallikrein 6 gene in some ovarian tumour samples. As chromosome 19q13 is nonrandomly rearranged in many human solid tumours including pancreatic carcinomas, astrocytomas, thyroid tumours, and ovarian cancers (Mitelman, 1994, pp 3067–3198), gene amplification is likely another mechanism for the elevated expression of hK6 in ovarian tumours.

The functional roles of kallikrein 6 and many other kallikreins have not yet been established. As they are secreted serine proteases, their actions through the degradation of the extracellular matrix may facilitate tumour cell spread. Alternatively, they may be part of an enzymatic cascade pathway that involves enzyme activation followed by proteolysis (Matrisian, 1999). The establishment of functional recombinant proteins and kallikrein-expressing cell lines will facilitate functional studies and provide information about the possible roles of these kallikreins in aberrant cell growth and cell invasion.

In summary, we have developed a novel monoclonal antibody to confirm the high expression levels of hK6 in ovarian tumour cells than in normal ovarian epithelial cells. High expression of hK6 protein in coexisting benign, borderline and invasive ovarian tumours was observed. The examined tumours also expressed high levels of kallikrein 6 mRNA. Southern blot and quantitative real-time PCR using genomic DNA suggested that gene amplification is one mechanism for the high expression of kallikrein 6 in ovarian tumours. The development of high-quality monoclonal antibodies facilitates the evaluation of biomarkers and allows further screening tool development for early detection of ovarian cancer.

## References

[bib1] Anisowicz A, Sotiropoulou G, Stenman G, Mok SC, Sager R (1996) A novel protease homolog differentially expressed in breast and ovarian cancer. Mol Med 2: 624–636 8898378 PMC2230195

[bib2] Ausubel FM, Brent R, Kingston RE, Moore DD, Seidman JG, Smith JA, Struhl K (eds) (1988) Current Protocols in Molecular Biology. New York: John Wiley & Sons

[bib3] Bast RC, Boyer CM, Olt GJ, Berchuck A, Soper JT, Clarke-Pearson D, Xu FJ, Ramakrishnan S (1990) Identification of marker for early detection of epithelial ovarian cancer. In Ovarian Cancer Biological and Therapeutic Challenges, Sharp F, Mason WP, Leake RE (eds). pp 265–275, London, England: Chapman & Hall Medical

[bib4] Bast RC, Hunter V, Knapp RC (1987) Pros and cons of gynecological tumor markers. Cancer 60: 1984–1992 2443235 10.1002/1097-0142(19901015)60:8+<1984::aid-cncr2820601510>3.0.co;2-w

[bib5] Bast RC, Xu FJ, Yu YH, Barnhill S, Zhang Z, Mills GB (1998) CA125: the past and the future. Int J Biol Markers 13: 179–187 10228898 10.1177/172460089801300402

[bib6] Bell DA, Scully RE (1994) Early *de novo* ovarian carcinoma. A study of fourteen cases. Cancer 73: 1859–1864 8137211 10.1002/1097-0142(19940401)73:7<1859::aid-cncr2820730714>3.0.co;2-l

[bib7] Berek JS, Bast RC (1995) Ovarian cancer screening. The use of serial complementary tumor markers to improve sensitivity and specificity for early detection. Cancer 76: 2092–2096 8635006 10.1002/1097-0142(19951115)76:10+<2092::aid-cncr2820761331>3.0.co;2-t

[bib8] Catalona WJ, Smith DS, Ratliff TL, Dodds KM, Coplen DE, Yuan JJ, Petros JA, Andriole GL (1991) Measurement of prostate-specific antigen in serum as a screening test for prostate cancer. N Engl J Med 324: 1156–1161 1707140 10.1056/NEJM199104253241702

[bib9] Chen L-M, Karlan EY (1998) Early detection and risk reduction for familial gynecologic cancers. Clin Obstet Gynecol 41: 200–214 9504236 10.1097/00003081-199803000-00025

[bib10] Cohen P, Graves HCB, Peehl DM, Kamarei M, Giudice LC, Rosenfeld RG (1992) Prostate-specific antigen is an insulin-like growth factor binding protein-3 protease found in seminal plasma. J Clin Endocrinol Metab 75: 1046–1053 1383255 10.1210/jcem.75.4.1383255

[bib11] Diamandis EP, Yousef GM, Soosaipillai AR, Bunting P (2000) Human kallikrein 6 (zyme/protease M/neurosin): a new serum biomarker of ovarian carcinoma. Clin Biochem 33: 579–583 11124344 10.1016/s0009-9120(00)00182-x

[bib12] Huang K-C, Rao PH, Lau CC, Heard E, Ng S-K, Brown C, Mok SC, Berkowitz RS, Ng S-W (2002) Relationship of XIST expression and responses of ovarian cancer to chemotherapy. Mol Cancer Ther 1: 769–776 12492109

[bib13] Junor EJ, Hole DJ, Gillis CR (1994) Management of ovarian cancer-referral to a multidisciplinary team matters. Br J Cancer 70: 363–370 8054286 10.1038/bjc.1994.307 PMC2033481

[bib14] Kim H, Scorilas A, Katsaros D, Yousef GM, Massobrio M, Fracchioli S, Piccinno R, Gordini G, Diamandis EP (2001) Human kallikrein gene 5 (KLK5) expression is an indicator of poor prognosis in ovarian cancer. Br J Cancer 84: 643–650 11237385 10.1054/bjoc.2000.1649 PMC2363783

[bib15] Mackey SE, Creasman WT (1995) Ovarian cancer screening. J Clin Oncol 13: 783–793 7884436 10.1200/JCO.1995.13.3.783

[bib16] Matrisian LM (1999) Cancer biology: extracellular proteinases in malignancy. Curr Biol 9: R776–R778 10531025 10.1016/S0960-9822(00)80011-1

[bib17] Mettlin C, Jones G, Averette H, Gusberg SB, Murphy GP (1993) Defining and updating the American Cancer Society Guidelines for the cancer-related checkup: prostate and endometrial cancers. CA Cancer J Clin 43: 42–46 8422604 10.3322/canjclin.43.1.42

[bib18] Mitelman F (1994) Catalog of Chromosome Aberrations in Cancer, 5th edn, pp 3067–3198, New York: Wiley-Liss

[bib19] Ozols RF (2002) Future directions in the treatment of ovarian cancer. Semin Oncol 29: 32–42 10.1053/sonc.2002.31594 11840418

[bib20] Powell DE, Puls L, van Nagell J (1992) Current concepts in epithelial ovarian tumors: does benign to malignant transformation occur? Hum Pathol 23: 846–847 1644430 10.1016/0046-8177(92)90393-h

[bib21] Ryuko K, Miura H, Abu-Musa A, Iwanari O, Kitao M (1992) Endosalpingiosis in association with ovarian surface papillary tumor of borderline malignancy. Gynecol Oncol 46: 107–110 1634129 10.1016/0090-8258(92)90205-w

[bib22] Schachter M. (1980) Kallikreins (kininogenases) – a group of serine proteases with bioregulatory actions. Pharmacol Rev 31: 1–17 94166

[bib23] Schwartz PE, Taylor KJ (1995) Is early detection of ovarian cancer possible? Ann Med 27: 519–528 8541026 10.3109/07853899509002463

[bib24] Scully RE, Bell DA, Abu-Jawdeh GM (1992) Update on early ovarian cancer and cancer developing in benign ovarian tumors. In Ovarian Cancer 3, Sharp F, Mason P, Blackett T, Berek J (eds). pp 139–144, London, England: Chapman & Hall Medical

[bib25] Taylor KJW, Schwartz PE (1994) Screening for early ovarian cancer. Radiology 192: 1–10 8208918 10.1148/radiology.192.1.8208918

[bib26] Tsao SW, Mok SC, Fey EG, Fletcher JA, Wan TS, Chew EC, Muto MG, Knapp RC, Berkowitz RS (1995) Characterization of human ovarian surface epithelial cells immortalized by human papilloma viral oncogenes (HPV-E6E7 ORFs). Exp Cell Res 218: 499–507 7796885 10.1006/excr.1995.1184

[bib27] Underwood LJ, Tanimoto H, Wang Y, Shigemasa K, Parmley TH, O'Brien TJ (1999) Cloning of tumor-associated differentially expressed gene-14, a novel serine protease overexpressed by ovarian carcinoma. Cancer Res 59: 4435–4439 10485494

[bib28] Yiu GK, Chan WY, Ng S-W, Chan PS, Cheung KK, Berkowitz RS, Mok SC (2001) SPARC (Secreted protein acidic and rich in cysteine) induces apoptosis in ovarian cancer cells. Am J Pathol 159: 609–622 11485919 10.1016/S0002-9440(10)61732-4 PMC1850537

[bib29] Young CYF, Andrews PE, Tindall DJ (1995) Expression and androgenic regulation of human prostate-specific kallikreins. J Androl 16: 97–99 7559150

[bib30] Yousef GM, Diamandis EP (2001) The new human tissue kallikrein gene family: structure, function, and association to disease. Endocr Rev 22: 184–204 11294823 10.1210/edrv.22.2.0424

[bib31] Yousef GM, Bharaj BS, Yu H, Poulopoulos J, Diamandis EP (2001) Sequence analysis of the human kallikrein gene locus identifies a unique polymorphic minisatellite element. Biochem Biophys Res Comm 285: 1321–1329 11478802 10.1006/bbrc.2001.5321

[bib32] Yousef GM, Polymeris ME, Yacoub GM, Scorilas A, Soosaipillai A, Popalis C, Fracchioli S, Katsaros D, Diamandis EP (2003) Parallel overexpression of seven kallikrein genes in ovarian cancer. Cancer Res 63: 2223–2227 12727843

