# Characterization of human kallikrein 6/prostease M expression in ovarian cancer

**DOI:** 10.1038/sj.bjc.6602508

**Published:** 2005-03-08

**Authors:** X Ni, W Zhang, K-C Huang, Y Wang, S-K Ng, S C Mok, R S Berkowitz, S-W Ng

**Correction to:** *British Journal of Cancer* (2004) **91**, 725–731. doi:10.1038/sj.bjc.6602041

With reference to the above article published in the British Journal of Cancer, the authors wish to make the following statement:

Although one of us participated in the work that resulted in the first publication about kallikrein 6/protease M, we were not physically involved in the first cloning of kallikrein 6 cDNA. Nevertheless, this does not have any influence on the conclusions of this article.

